# Pathobiology and innate immune responses of gallinaceous poultry to clade 2.3.4.4A H5Nx highly pathogenic avian influenza virus infection

**DOI:** 10.1186/s13567-019-0704-5

**Published:** 2019-11-01

**Authors:** Kateri Bertran, Mary J. Pantin-Jackwood, Miria F. Criado, Dong-Hun Lee, Charles L. Balzli, Erica Spackman, David L. Suarez, David E. Swayne

**Affiliations:** 1 Exotic and Emerging Avian Viral Diseases Research Unit, Southeast Poultry Research Laboratory, U.S. National Poultry Research Center, Agricultural Research Service, U.S. Department of Agriculture, Athens, GA 30605 USA; 2 Present Address: IRTA, Centre de Recerca en Sanitat Animal (CReSA, IRTA-UAB), Campus de la Universitat Autònoma de Barcelona, 08193 Bellaterra, Barcelona, Spain; 3 Present Address: Department of Pathobiology & Veterinary Science, University of Connecticut, Storrs, CT 06269 USA; 4 Present Address: Battelle National Biodefense Institute, National Biodefense Analysis and Countermeasures Center, 8300 Research PI, Fort Detrick, MD 21702 USA

## Abstract

In the 2014–2015 Eurasian lineage clade 2.3.4.4A H5 highly pathogenic avian influenza (HPAI) outbreak in the U.S., backyard flocks with minor gallinaceous poultry and large commercial poultry (chickens and turkeys) operations were affected. The pathogenesis of the first H5N8 and reassortant H5N2 clade 2.3.4.4A HPAI U.S. isolates was investigated in six gallinaceous species: chickens, Japanese quail, Bobwhite quail, Pearl guinea fowl, Chukar partridges, and Ring-necked pheasants. Both viruses caused 80–100% mortality in all species, except for H5N2 virus that caused 60% mortality in chickens. The surviving challenged birds remained uninfected based on lack of clinical disease and lack of seroconversion. Among the infected birds, chickens and Japanese quail in early clinical stages (asymptomatic and listless) lacked histopathologic findings. In contrast, birds of all species in later clinical stages (moribund and dead) had histopathologic lesions and systemic virus replication consistent with HPAI virus infection in gallinaceous poultry. These birds had widespread multifocal areas of necrosis, sometimes with heterophilic or lymphoplasmacytic inflammatory infiltrate, and viral antigen in parenchymal cells of most tissues. In general, lesions and antigen distribution were similar regardless of virus and species. However, endotheliotropism was the most striking difference among species, with only Pearl guinea fowl showing widespread replication of both viruses in endothelial cells of most tissues. The expression of IFN-γ and IL-10 in Japanese quail, and IL-6 in chickens, were up-regulated in later clinical stages compared to asymptomatic birds.

## Introduction

The H5 A/goose/Guangdong/1/1996 (Gs/GD) lineage of highly pathogenic avian influenza (HPAI) virus has spread across multiple continents, affecting wild birds, poultry, and humans [1]. In 2014, Gs/GD lineage clade 2.3.4.4 Group A, also described as Buan2-like or icA, spread across Asia, Europe, and North America [2]. The initial detection of this viral lineage into North America was a reassortant H5N2 with five Eurasian avian influenza (AI) virus gene segments (including the H5 clade 2.3.4.4 hemagglutinin) and three North American wild bird lineage low pathogenic AI (LPAI) virus gene segments [3, 4] detected in November 2014 in British Columbia, Canada. Concurrently, an H5N8 HPAI virus with all 8 gene segments of Eurasian origin and the reassortant H5N2 HPAI virus were detected in a captive-reared gyrfalcon (*Falco rusticolus*) and a wild Northern pintail duck (*Anas acuta*), respectively, in Washington state, U.S. Over the next 7 months, the U.S. poultry industry experienced an unprecedented outbreak caused by these H5 HPAI viruses, with more than 7.5 million turkeys and 42.1 million chickens having died or culled during the control program [5] and widespread bans on exports of U.S. poultry and poultry products [6].

During the outbreak, virus was detected in 21 backyard flocks that included: (i) minor gallinaceous poultry species (quail, guinea fowl, pheasants, partridges, and grouse) on the same premise; (ii) different breeds of chickens and turkeys; and (iii) domestic ducks and geese [5, 7]. Minor gallinaceous poultry are commonly raised in small commercial outdoor operations and backyards, or are sometimes marketed in temporary or permanent live poultry markets (LPMs) [8], which highlights their importance as intermediary hosts in virus transmission under poor biosecurity conditions. Globally, two facts support their importance in AI epidemiology: (i) outdoor-raised systems often have higher potential of AI virus exposure from wild waterfowl, contributing to the increase of AI outbreaks and their impact [9]; and (ii) LPMs, with tendency for prevalent AI virus [10], contain a wide variety of live poultry and non-poultry species, providing the ideal environment for introduction, maintenance, and adaptation of viruses, as well as potential conditions for zoonotic transmission [11–13]. Although there is no direct epidemiological link between minor gallinaceous poultry from backyard flocks and chickens and turkeys from commercial farms in these U.S. clade 2.3.4.4A virus outbreaks [14], minor poultry have been identified or suggested as key link species in other outbreaks [15–18]. Moreover, the game bird poultry industry and backyard flocks in both developed and developing countries are known to suffer from HPAI epidemics [19].

Gallinaceous species infected with clade 2.3.4.4 viruses generally exhibit clinical disease, mortality, and pathological features that are indicative of HPAI virus infection [2], although several studies have pointed out that clade 2.3.4.4 H5 reassortants have reduced virulence compared to the parental Gs/GD H5N1 virus [2, 4, 20]. Lee et al. examined the pathogenicity of clade 2.3.4.4 viruses in Japanese quail and showed severe clinical disease, virus shedding, and contact transmission following challenge with 6 log_10_ EID_50_ of Korean Group A H5N8 virus [21]. Prior to the emergence of clade 2.3.4.4 viruses, numerous outbreaks of both LPAI and HPAI viruses had been reported in species such as Japanese quail, Pearl guinea fowl, and Ring-necked pheasants [19, 22]. Some studies suggest that certain gallinaceous species like Ring-necked pheasants and Japanese quail are more susceptible to LPAI viruses from free-living aquatic birds than chickens and turkeys [19, 23–28]. Others show that Japanese quail and European quail (*Coturnix c. coturnix*) may support the replication of almost all LPAI virus subtypes [26, 29]. Japanese quail are recognized as mixing vessels for avian and mammalian viruses [30–33] and facilitate the adaptation of duck viruses to chicken [34–36]. In addition, several studies have proven that HPAI viruses are able to infect and cause lesions and death in many gallinaceous species under experimental conditions [19, 37, 38]. Collectively, these findings highlight the relevance of avian species other than chickens, turkeys, and domestic ducks in the epidemiology of AI in small farming operations, village poultry, and LPMs.

Recently, we experimentally confirmed that the first U.S. Eurasian H5N8 and reassortant H5N2 clade 2.3.4.4A HPAI viruses lacked adaptation to chickens, i.e. 4.4 and 5.7 log_10_ mean bird infectious doses (BID_50_), respectively [39], but were more adapted to minor gallinaceous poultry, i.e. < 3.7 log_10_ BID_50_ for direct infection [40]. In addition, these higher BID_50_ required to produce chicken infections also resulted in greatly reduced contact transmission. Here we present the pathology results from our previously published infectivity studies [39, 40]. Specifically, we examined the severity and distribution of gross and microscopic lesions in experimentally infected birds and identified the organs and cell types with AI virus replication. In addition, we analyzed the innate immune response in chickens and Japanese quail at different clinical stages.

## Materials and methods

### Viruses

The influenza A isolates A/Gyrfalcon/Washington/40188-6/2014 (H5N8) and A/Northern pintail/Washington/40964/2014 (H5N2) were used as challenge viruses. These were the first two HPAI isolates from the U.S. outbreak and they are considered representative of the initial AI viruses from wild waterfowl introduction of both the Eurasian lineage H5N8 viruses and the reassortant Eurasian/North American lineage H5N2 viruses, respectively [40]. The viruses were propagated and titrated by allantoic sac inoculation of 9–10 day-old embryonated chicken eggs by standard methods [41].

### Birds and housing

Six species of the order Galliformes were utilized: specific pathogen free White Leghorn chickens (*Gallus domesticus*; Southeast Poultry Research Laboratory [SEPRL], Athens, GA, USA), Japanese quail (*Coturnix c. japonica*; McMurray Hatchery, Webster City, IA, USA), Bobwhite quail (*Colinus virginianus*; M&M Quail Farm Inc., Gillsville, GA, USA), Pearl guinea fowl (*Numida meleagris*; McMurray Hatchery), Chukar partridges (*Alectoris chukar*; McMurray Hatchery), and Ring-necked pheasants (*Phasianus colchicus*; McMurray Hatchery). All birds were inoculated at 4 weeks of age. Prior to inoculation, 30–50% of birds were randomly sampled and confirmed negative for current infection with or previous exposure to AI virus [42, 43]. Each experimental group was housed separately in negative-pressure isolators with HEPA-filtered inlet air. Birds had ad libitum access to feed and water. All procedures were performed according to the requirements of the protocol approved by the Institutional Laboratory Animal Care and Use Committee.

### Experimental design and sampling

Bird inoculation and sampling were performed as previously described [39, 40]. Briefly, each species was divided into three groups: H5N2 virus inoculated group, H5N8 virus inoculated group, and sham-inoculated group (10 to 17 birds/virus group and 5 birds/sham group). Birds were inoculated intrachoanally with approximately 6 log_10_ mean egg infectious doses (EID_50_) of H5N2 or H5N8 virus, or sterile allantoic fluid. This challenge dose was necessitated for comparison with previous HPAI pathogenesis studies [21, 25, 44–48]. Clinical signs were monitored twice a day during the first 4 days post-challenge (dpc) and daily thereafter. Two birds from each species exposed to each virus and showing severe clinical signs or found dead were necropsied at 2 and 3 dpc, except for chickens and Japanese quail that were necropsied at four time points based on clinical progression, as previously described [39, 40]: asymptomatic (twice, at 18 and 24 h post-challenge [hpc]), listless (showing mild to moderate clinical signs), and moribund or dead. One sham-inoculated bird of each species was euthanized and necropsied at the first and the last necropsy time points. At time of necropsy, portions of nasal cavity, brain, thymus, trachea, lung, proventriculus, duodenum, pancreas, jejunum-ileum, spleen, kidney, adrenal gland, gonad, liver, skeletal muscle, comb, and heart were collected in 10% buffered formalin (Thermo Fisher Scientific, Waltham, MA, USA) for histopathologic evaluation. Severely sick birds were euthanized. At 10 dpc, surviving birds were bled to evaluate antibody titers and euthanized.

### Histopathology and immunohistochemistry

Tissues in 10% formalin were processed for routine hematoxylin and eosin (HE) staining and immunohistochemical (IHC) staining using a mouse-derived monoclonal antibody (P13C11, developed at SEPRL) specific for type A influenza virus nucleoprotein [39, 44].

### Quantification of innate immune response genes

The mRNA expression of genes representative of different innate pathways, including type 1 interferon (IFN) (IFN-α), type 2 IFN (IFN-γ), Th1-type cytokine (interleukin (IL)-12, IL-18), Th2-type cytokine (IL-10), pro-inflammatory cytokine (IL-6), and toll-like receptor (TLR-7), was quantified from formalin-fixed paraffin-embedded (FFPE) lung and spleen tissues from chickens and Japanese quail necropsied at different clinical stages of infection. The FFPE tissue sections and RNA extraction were performed as previously described [49] with modifications. Briefly, ten 10-μm-thick sections were collected and deparaffinized, total RNA was extracted using the RNeasy FFPE Kit (Qiagen, Germantown, MD, USA), and RNA concentration and purity were measured on a NanoDrop 1000 Spectrophotometer (Thermo Fisher Scientific). The cDNA was synthetized with the High Capacity cDNA Reverse Transcription Kit (Thermo Fisher Scientific) using approximately 500 ng of RNA and following the manufacturer’s protocol and thermal cycling conditions. The mRNA expressions of the aforementioned genes were quantified by quantitative real time PCR (qRRT-PCR) using gene specific primers and conditions previously described [50, 51] with modifications. Briefly, qRRT-PCR was performed on a 7500 FAST Real-time PCR System (Applied Biosystems, Foster City, CA, USA). The qRRT-PCR reaction mixture contained 1.0 μL of sample cDNA, 0.1 to 0.5 μL of forward and reverse primers (1 to 5 μM each, standardized for efficient detection of target gene and absence of dimers) (Additional file 1), 5 μL of KAPA SYBR FAST MasterMix (KAPA Biosystems, Wilmington, MA, USA), and nuclease free water for a final reaction volume of 10.0 μL. The thermal profile consisted of one cycle for 3 min of polymerase activation at 95 °C, followed by 45 cycles of PCR at 95 °C for 10 s and specific annealing temperature of 60 °C for 30 s. After the completion of amplification step, the dissociation (melting) curve was performed at 95 °C for 10 s and 60 °C for 30 s. The relative expression of each target gene was normalized using the housekeeping gene *β*-*actin* (Additional file 1). The expression of *β*-*actin* was constant within each species regardless of clinical stage and tissue. The relative quantification of gene expression was done by the 2 − ΔΔct formula and expressed as fold change in infected birds compared to sham (negative control) birds. Results from H5N2 and H5N8 virus infected birds were pooled due to similar mRNA expression levels for all the genes. Similarly, results from moribund and dead birds were pooled due to similar mRNA expression levels for all the genes. After normalization with *β*-actin, gene expression results in infected birds were analyzed based on species, tissue, and clinical stage and compared to sham birds. Our data had a non-parametric distribution and was analyzed with Kruskal–Wallis test and Dunn’s Multiple Comparison Test using Prism 7 (GraphPad software, San Diego, CA, USA). A *p* value of < 0.05 was considered to be significant.

## Results

### Clinical signs, mortality, and gross lesions

Previously, we determined that intrachoanal inoculation of 6 log_10_ EID_50_ of either H5N2 or H5N8 virus caused 80–100% mortality in the six gallinaceous species [39, 40], with the exception of H5N2 virus that caused 60% mortality in chickens [39] (Table 1). Mean death times (MDTs) ranged from 2.5 to 5.2 days and were not significantly different among them [39, 40] (Table 1). The surviving birds were considered uninfected based on lack of clinical disease and lack of HA antibodies at the end of the experiment [39, 40]. Among the infected birds, clinical signs and gross lesions are described in detail for chickens [39] and the other gallinaceous species [40] elsewhere.

**Table 1:** **Summary results of gallinaceous poultry challenged with 6 log**_**10**_ **EID**_**50**_ **of A/northern pintail/Washington/40964/2014 (H5N2) and A/gyrfalcon/Washington/40188-6/2014 (H5N8).** Data from [39, 40]

Species	H5N2			H5N8		
	Mortality^a^	MDT^b^	Log_10_ BID_50_^c^	Mortality^a^	MDT^b^	Log_10_ BID_50_^c^
Chicken	3/5 (60%)	3.0	5.7	5/5 (100%)	4.1	4.4
Japanese quail	4/5 (80%)	2.8	3.7	4/5 (80%)	2.5	3.2
Bobwhite quail	7/7 (100%)	4.7	< 2	8/8 (100%)	4.9	< 2
Pearl guinea fowl	5/5 (100%)	2.8	3.0	5/5 (100%)	3.8	3.0
Chukar partridge	8/8 (100%)	4.1	3.6	7/8 (87.5%)	5.2	3.6
Ring-necked pheasant	8/8 (100%)	4.7	3.4	8/8 (100%)	4.8	3.0

^a^#dead birds/total (%)

^b^MDT, mean death time: #dead birds × dpc/total dead birds (expressed as dpc). MDTs were not statistically different among species or between viruses

^c^BID_50_, mean bird infectious dose

### Microscopic findings

**Figure 1:**
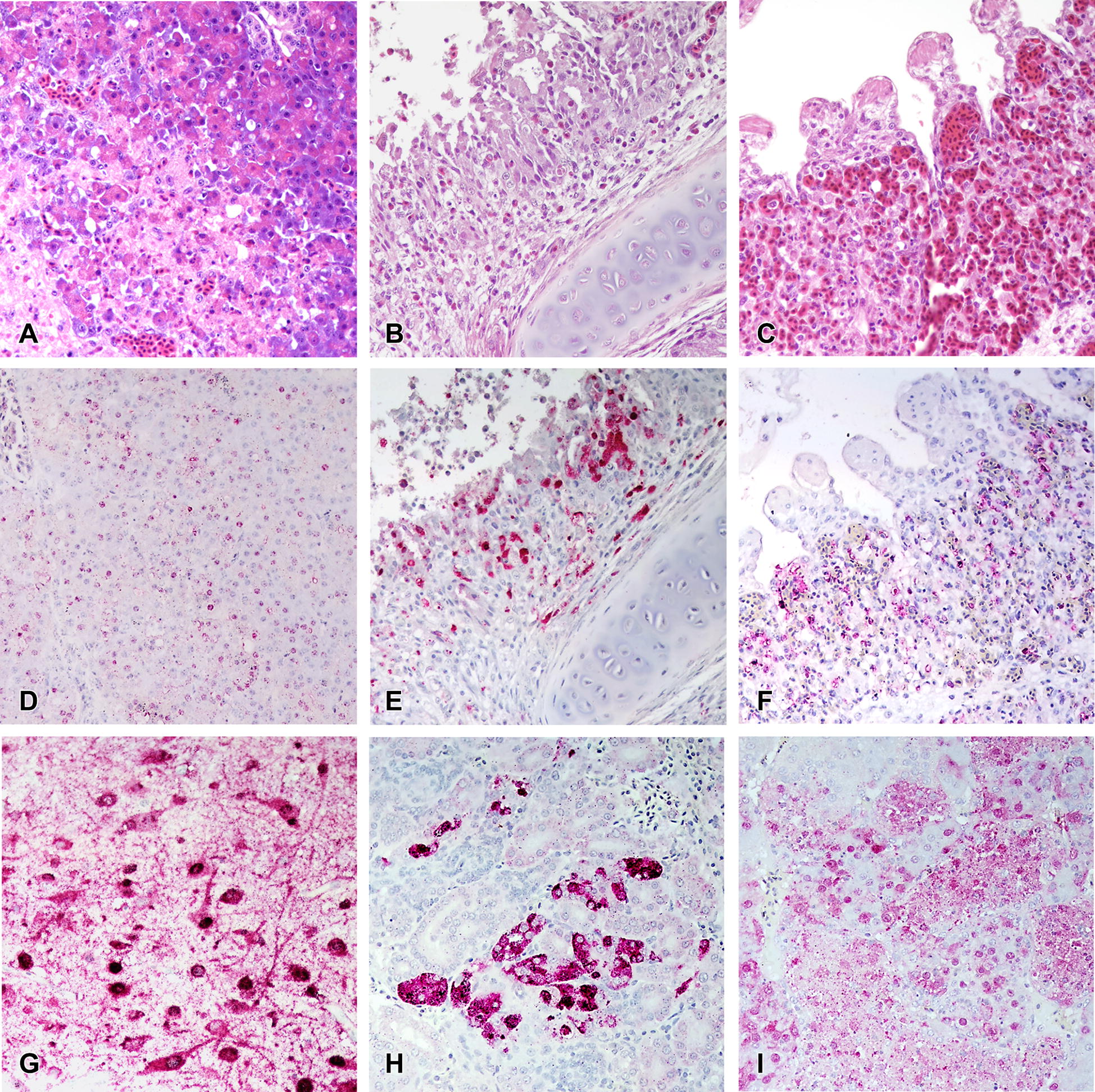
**Histological lesions in gallinaceous following experimental infection with clade 2.3.4.4A HPAI viruses.** A/Northern pintail/Washington/40964/2014 (H5N2); A/Gyrfalcon/Washington/40188-6/2014 (H5N8). Magnification ×40. Japanese quail, listless (3 dpc), H5N2 virus, pancreas, HE staining (**A**) and IHC staining (**D**). Bobwhite quail, 3 dpc, H5N2 virus, respiratory epithelium, HE staining (**B**) and IHC staining (**E**). Pearl guinea fowl, 2 dpc, H5N8 virus, lung (endothelium), HE staining (**C**) and IHC staining (**F**). Ring-necked pheasant, 3 dpc, H5N2 virus, cerebrum, IHC staining (**G**). Ring-necked pheasant, 3 dpc, H5N2 virus, kidney, IHC staining (**H**). Chukar partridge, 3 dpc, H5N8 virus, adrenal gland, IHC staining (**I**).

Multifocal areas of necrosis, sometimes accompanied by heterophilic or lymphoplasmacytic inflammatory infiltrate, with viral antigen were widespread in the parenchymal cells of most tissues (Tables 2, 3, 4 and Figure 1). In general, asymptomatic or listless chickens and Japanese quail did not show significant histopathological lesions or antigen staining, with some exceptions like severe vacuolation and necrosis of the pancreatic acinar epithelium of a listless H5N2 virus infected Japanese quail (Figure 1A) and nucleoprotein-positive pancreatic acinar cells (Figure 1D). More severe lesions and widespread viral staining were observed in moribund and dead birds, especially in lung, heart, brain, pancreas, spleen, and adrenal gland.

**Table 2:** **Microscopic lesions and viral antigen distribution in gallinaceous inoculated with A/Northern pintail/Washington/40964/2014 (H5N2) HPAI virus**

	Chicken		Japanese quail		Bobwhite quail		Pearl guinea fowl		Chukar partridge		Ring-necked pheasant	
Clinical stage or day post-challenge^a^	Asymptomatic Listless Moribund/dead		Asymptomatic Listless Moribund/dead		2d 2d 3d 3d		2d 2d		2d 2d 3d 3d		2d 2d 3d 3d	

Tissue	Histo score^b^	IHC score^c^	Histo score^b^	IHC score^c^	Histo score^b^	IHC score^c^	Histo score^b^	IHC score^c^	Histo score^b^	IHC score^c^	Histo score^b^	IHC score^c^
Nasal cavity	− + ++	− + +	− − −	− − −	++ − ++ +++	+++ +++ +++ +++	− −	+++ +++	− − − −	− − − ++	− − − −	− − − +
Trachea	− − −	− − −	− − −	− − −	− nt − −	− nt − −	− nt	− nt	− − − −	− + − −	− − nt ++	− − nt +
Lung	− − ++	− − ++	− ++ −	− +++ +	− − − −	+++ + +++ +	+++ +++	+++ +++	− ++ +++ +++	− +++ +++ +++	− ++ ++ ++	− +++ +++ +++
Comb	− + +++	− + +++	nt nt nt	nt nt nt	− − − −	+++ +++ − −	− −	+++ +++	− − − −	− − − ++	− − − −	− − + +
Heart	− − +++	− + +++	− − ++	− − +++	+ + ++ +	+++ ++ +++ ++	− −	++ ++	− + + ++	− +++ +++ +++	− + ++ +++	− +++ +++ +++
Brain	− − ++	− − +++	− − +	− − +++	− − − −	+++ ++ +++ ++	− −	+++ +++	− − − +	− ++ ++ +++	− + − +	− + +++ +++
Proventriculus	− − +	− − +	nt nt nt	nt nt nt	− − − −	− − − −	− nt	− nt	− − − −	− + − +	− − − +	− − − ++
Intestine	− − +	− − +	− − −	− − −	− − + −	− − + −	− −	++ +	− − − −	− − − −	− − − −	− − − ++
Pancreas	− − +	− − −	− + +++	− ++ +++	− − ++ −	− − +++ −	− −	++ +	− − − +	− + ++ ++	− − − +++	− − + +++
Liver	− − −	− + +	− − −	− − −	− − + −	− − +++ −	− −	+++ ++	− − − −	− ++ − +	− − − −	− +++ − ++
Spleen	− + ++	− + ++	− + −	− + +	+++ ++ + nt	++ +++ ++ nt	− −	+++ +++	− + − +	− +++ − ++	− ++ + ++	− +++ +++ +++
Thymus	− + ++	− − ++	− − nt	− − nt	− + + +	− + ++ ++	− −	+++ ++	− − − +	− + +++ ++	− + + +	− ++ ++ +++
Cloacal bursa	− − ++	− − ++	+ + +	− − −	+++ +++ ++ ++	++ + +++ ++	nt +	nt +	− − − +	− + + −	− ++ ++ ++	− − ++ +++
Kidney	− − +	− − ++	− − −	− − −	− − − −	− − − −	− −	++ +	− − − +	− ++ ++ ++	− − + +	− − +++ +++
Gonad	− − −	− − +	− − +	− − +++	− nt nt nt	+ nt nt nt	nt nt	nt nt	− + nt +	− +++ nt ++	nt nt nt nt	nt nt nt nt
Adrenal gland	− − +	− − ++	− − +	− − +++	− nt nt nt	− nt nt nt	nt nt	nt nt	− nt nt +++	− nt nt +++	− nt +++ nt	− nt +++ nt
Skeletal muscle	− − −	− − −	− − −	− − −	− − − −	− − + −	− −	++ +	− − − −	− − − −	− − nt nt	− ++ nt nt

nt: no tissue.

^a^For chicken and Japanese quail, scores represent the mean of the birds sampled at each clinical stage: asymptomatic (18 and 24 hpc), listless, and moribund/dead.

^b^Histopathology score of lesions in HE staining: −, no lesions; +, mild; ++, moderate; +++, severe.

^c^Immunohistochemical (IHC) staining: −, no antigen staining; +, infrequent; ++, common; +++, widespread.

**Table 3:** **Microscopic lesions and viral antigen distribution in gallinaceous inoculated with A/Gyrfalcon/Washington/40188-6/2014 (H5N8) HPAI virus**

	Chicken		Japanese quail		Bobwhite quail		Pearl guinea fowl		Chukar partridge		Ring-necked pheasant	
Clinical stage or day post-challenge^a^	Asymptomatic Listless Moribund/dead		Asymptomatic Listless Moribund/dead		2d 2d 3d 3d		2d 2d 3d		2d 2d 3d 3d		2d 2d 3d 3d	

Tissue	Histo score^b^	IHC score^c^	Histo score^b^	IHC score^c^	Histo score^b^	IHC score^c^	Histo score^b^	IHC score^c^	Histo score^b^	IHC score^c^	Histo score^b^	IHC score^c^
Nasal cavity	− − +	− − +	− − +	− ++ ++	++ ++ ++ +	+++ +++ +++ +++	− − −	+++ +++ ++	− + − −	− + + −	− − − −	− − ++ ++
Trachea	− − +	− − +	− − −	− − −	− − − −	− ++ − −	− − −	− − −	− + − −	− − + −	− − + −	− − ++ +
Lung	− − ++	− − +	− ++ +	− +++ ++	− ++ + −	+ +++ ++ +	+++ +++ +++	+++ +++ +++	+++ + ++ +	+++ ++ ++ ++	− − ++ +	++ − +++ ++
Comb	− − +++	− − +++	nt nt nt	nt nt nt	− − + +	++ ++ +++ ++	− − +	++ ++ ++	− − − −	+ − ++ −	nt − nt nt	nt − nt nt
Heart	− − +++	− − +++	− + +++	− +++ +++	+ + + ++	− +++ +++ +++	− − +	+++ +++ +++	+ + ++ ++	++ ++ +++ +++	+ − ++ ++	++ ++ +++ +++
Brain	− − +	− − +++	− + +++	− +++ +++	− − + −	− + +++ +++	− − −	+++ +++ +++	− − − −	++ + +++ ++	− − ++ ++	+ ++ +++ +++
Proventriculus	− − −	− − +	nt nt nt	nt nt nt	− − − −	− + − −	− − −	++ +++ −	− − − −	− − − −	− − − −	− − ++ −
Intestine	− − −	− − −	− − −	− − −	− − − −	− − − −	nt − −	nt − −	− − − −	+ − − −	− − − −	− − ++ −
Pancreas	− − +	− − +	− ++ +++	− +++ +++	− − ++ +	− − ++ +	− − ++	− + +	+ − +++ +++	++ − +++ +++	− − +++ +	− − +++ ++
Liver	− − −	− − +	− − −	− − −	− − − −	− + + −	− − −	+ ++ −	+ − − −	++ − − −	− − − ++	− − ++ −
Spleen	− − +	− − +	− ++ +	− ++ −	+ + ++ +	− ++ +++ ++	++ + +	+++ +++ ++	+ − − −	− − + −	+ + − −	− − +++ −
Thymus	− − +	− − +	− nt −	− nt −	+ − + nt	− − − nt	− − +	− − +	− − + −	+ − + −	− − − −	+ − +++ +++
Cloacal bursa	− − ++	− − +	− nt ++	− nt −	+ − ++ ++	− − + ++	+ − +	++ − −	++ − + −	+ − − +	+ − ++ ++	− − ++ −
Kidney	− − +	− − +	− − −	− − -	− nt − −	− nt + −	− − +	++ ++ ++	+ − + +	++ ++ ++ ++	− + ++ ++	− ++ +++ +++
Gonad	− − +	− − +	− − ++	− − ++	nt nt nt nt	nt nt nt nt	nt nt nt	nt nt nt	− − − −	− − − −	− nt − +	− nt ++ ++
Adrenal gland	− − ++	− − +	− + ++	− +++ +++	nt nt nt nt	nt nt nt nt	nt nt nt	nt nt nt	++ nt +++ +++	+++ nt +++ +++	++ ++ +++ +++	+++ +++ +++ +++
Skeletal muscle	− − −	− − +	− − +	− − −	− − − −	− − + −	− − −	+ + +	− − − −	− − − −	− − + +	− + +++ ++

nt: no tissue.

^a^For chicken and Japanese quail, scores represent the mean of the birds sampled at each clinical stage: asymptomatic (18 and 24 hpc), listless, and moribund/dead.

^b^Histopathology score of lesions in HE staining: −, no lesions; +, mild; ++, moderate; +++, severe.

^c^Immunohistochemical (IHC) staining: −, no antigen staining; +, infrequent; ++, common; +++, widespread.

**Table 4:** **Microscopic lesions and viral antigen distribution in gallinaceous inoculated with clade 2.3.4.4 H5Nx HPAI virus**

Tissue	Lesions	Cell types expressing virus antigen
Nasal cavity	Epithelial cell necrosis and desquamation, rhinitis, sinusitis, mononuclear cell infiltrate, heterophilic rhinitis	Vascular endothelial cells, nasal epithelial cells, nasal gland epithelium, mononuclear cells, bone marrow, autonomic nerves, buccal stratified epithelium, skeletal jaw muscle, feather pulp
Trachea	Focal necrosis with mild lymphoplasmacytic inflammatory infiltrate	Pseudostratified epithelial cells, vascular endothelial cells, sternotrachealis muscle
Lung	Severe interstitial pneumonia, edema, congestion, necrosis, monocytic infiltrate	Epithelium of air capillaries, mononuclear cells, necrotic debris
Comb	Edema, hemorrhages, necrosis	Vascular endothelial cells, mononuclear cells, necrotic debris, feather follicle epithelium, nerves in dermis, gland basilar cells, pili muscles
Heart	Focal necrosis of myocardiocytes	Myocardiocytes
Brain	Neuronal necrosis, gliosis, chromatolysis of Purkinje cell layer	Neurons, Purkinje cells, ependymal cells, glial cells, vascular endothelial cells
Proventriculus	Focal necrosis	Glandular and surface epithelium, nerves
Intestine	Lymphohistiocytic infiltration in submucosa	Mononuclear cells in lymphoid associated tissue, capillary endothelium and serosa
Pancreas	Degeneration of individual pancreatic acinar cells	Pancreatic acinar cells, duct cells, capillary endothelium
Liver	Focal necrosis with lymphoplasmacytic inflammatory infiltrate	Kupffer cells, hepatocytes, vascular endothelial cells, macrophages
Spleen	Multifocal areas of necrosis, hemorrhages, lymphoid depletion, hyperplasia of macrophage-phagocytic cells	Mononuclear cells, periarteriolar lymphoid sheaths, mononuclear phagocytic system
Thymus	Focal necrosis, lymphocyte depletion, apoptotic lymphocytes	Mononuclear cells, thymic epithelium in medullary area
Cloacal bursa	Lymphocyte necrosis and apoptosis, lymphocyte depletion, phagocytic hyperplasia	Mononuclear cells, medullary support cells, nerve ganglia
Kidney	Focal necrosis of tubular epithelium with lymphoplasmacytic inflammation	Tubular epithelial cells, glomerular cells
Gonad	Necrosis, focal interstitial necrosis with heterophilic infiltration	Tegument/interstitial tissue
Adrenal gland	Necrosis with mononuclear inflammatory infiltrate, heterophilic infiltrate	Corticotrophic and corticotropic cells
Skeletal muscle	Scattered necrotic fibers	Myocytes

Collectively, similar type and severity of histological lesions and antigen staining were observed with all species and both viruses, although some differences between the two viruses were observed (Tables 2 and 3). In the pancreas, lesions and staining were mild and infrequent in H5N2 virus infected birds, but were severe and widespread in H5N8 virus infected birds; in the thymus and cloacal bursa, the opposite was observed. A remarkable difference among species was observed in the kidney: Chukar partridges and Ring-necked pheasants (Figure 1H) displayed mild to moderate nephrosis and widespread staining, while chickens, Japanese quail, and Bobwhite quail had generally no lesions or staining, and lesions and staining in Pearl guinea fowl tissues were in between in extent. Another difference among species was found in nasal cavity, which was especially affected in Bobwhite quail (Figures 1B–E) and Pearl guinea fowl. Virus staining in vascular endothelial cells was infrequent in all the species examined except for Pearl guinea fowl, which showed extensive endothelial cell staining with both viruses. In particular, vascular endothelial cells expressed virus antigen in the following tissues: sheath arterioles of spleen of H5N2 virus inoculated Japanese quail; comb of H5N8 and nasal cavity of H5N2 virus inoculated Bobwhite quail; systemically in Pearl guinea fowl (Figure 1F); ovary of H5N8 virus inoculated Ring-necked pheasants; spleen and proventriculus of H5N2 and brain of H5N8 virus inoculated Chukar partridges.

### Immune gene expression profiles

**Figure 2:**
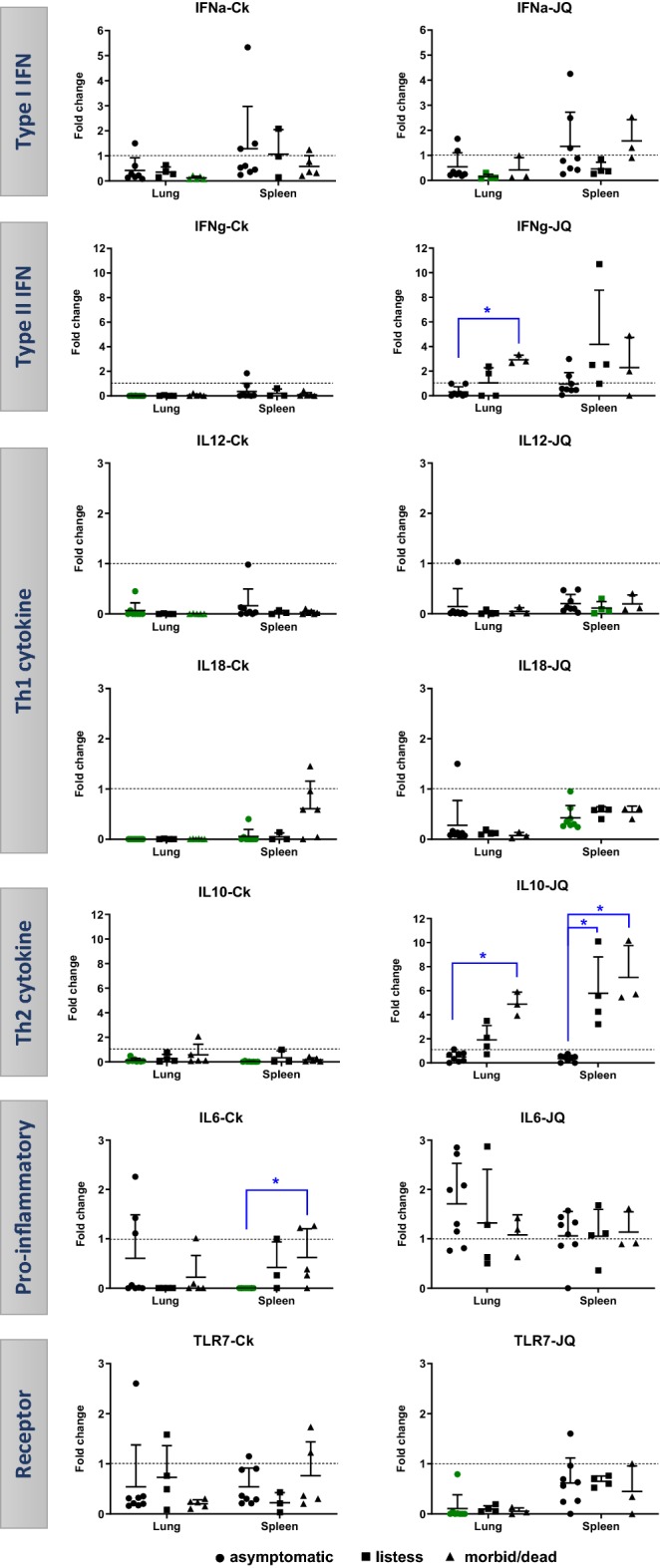
**Relative RNA expression of innate immune genes in chicken (Ck) and Japanese quail (JQ).** The fold change was calculated relative to levels of mRNA expression in infected birds compared to sham birds. Data plotted in green represents down-regulation (significantly lower than shams). Blue (*) shows significant differences between clinical stages ● asymptomatic, ■ listless, and ▲ moribund/dead. Significance *p* < 0.05.

The mRNA expression of innate immune response genes is summarized in Figure 2. The mRNA levels of IFN-γ were significantly up-regulated in lung of moribund/dead Japanese quail compared to asymptomatic birds. Similarly, the mRNA levels of IL-10 were significantly up-regulated in both lung and spleen of Japanese quail in later clinical stages compared to earlier clinical stages. In chickens, IL-6 was the only gene with significant variation among clinical stages, as its mRNA levels were significantly up-regulated in spleen of moribund/dead chickens compared to asymptomatic ones. Several genes were down-regulated in some clinical stages when compared to shams, like IFN-α, IL-12, and IL-18 for both species; IFN-γ and IL-10 for chickens; and TLR-7 for Japanese quail.

## Discussion

Since 2014, Gs/GD H5 HPAI clade 2.3.4.4 viruses have spread rapidly and globally by migratory aquatic birds and have evolved through reassortment with prevailing local LPAI viruses [2]. Under both experimental and natural conditions, a wide range of avian species including wild and domestic waterfowl, domestic poultry, and even zoo birds appear to be permissive for infection by and/or transmission with these viruses [2]. In the present study, the pathobiology of the first U.S. clade 2.3.4.4A HPAI viruses in the 2014–2015 outbreak was investigated in six gallinaceous species. In addition, innate immune responses to infection with these viruses were analyzed in chickens and Japanese quail.

We previously confirmed that these two viruses were poorly adapted to chickens, based on high mean chicken infectious dose (i.e. 4.4 and 5.7 log_10_ BID_50_) and poor transmissibility [39], but were more adapted to minor gallinaceous poultry (< 3.7 BID_50_) [40]. As expected with 6 log_10_ EID_50_ challenge dose, some chickens did not become infected, as evident by only 60% mortality with H5N2, and the survivors were confirmed as not being infected because they lacked clinical disease, virus shedding, and HA antibodies [39]. By contrast, high morbidity and mortality rates and severe pathobiology upon challenge with 6 log_10_ EID_50_ confirmed that these viruses were highly pathogenic for minor gallinaceous poultry [40].

In the present study, chickens and Japanese quail were necropsied at four time-points based on clinical progression. Similar type and severity of histological lesions and antigen staining were observed in both species and for both viruses at each clinical stage, but differences were observed between clinical stages. Asymptomatic and listless chickens and Japanese quail (necropsied from 18 hpc to 2–3 dpc) lacked histopathological lesions or antigen staining in the tissue samples tested, while moribund or dead birds (necropsied from 2 to 5 dpc) displayed more severe lesions and widespread viral staining in known HPAI virus-target tissues and cells [47]. Although rapidity of clinical progression is highly dependent on the HPAI virus strain [47], our findings are in line with previous studies showing lack of antigen staining when clinical signs are absent, but severe histopathological lesions and widespread antigen staining in moribund or dead gallinaceous birds [44, 52]. Our observations suggest that there is a short time window between the initial virus replication in epithelial cells of the respiratory tract and the subsequent viremia and dissemination to multiple organs, where the virus replicates in parenchymal cells [53]. The other four species were necropsied when showing severe clinical signs or found dead (at 2 and 3 dpc), with both viruses showing similar type and severity of histological lesions and antigen detection in tissues across species.

Among the few discrepancies observed in virus replication in tissues of different species, the vascular endothelium showed the most remarkable variations. We observed that chickens, Japanese quail, Bobwhite quail, Chukar partridges, and Ring-necked pheasants lacked widespread virus replication in capillary endothelial cells at any clinical stage, with consequent lack of severe edematous and hemorrhagic lesions. In contrast, Pearl guinea fowl presenting severe clinical signs or found dead had endothelial cell staining for both viruses in capillary endothelium of almost all tissues. Previous studies have shown that endotheliotropism is common in gallinaceous poultry infections with HPAI viruses [46, 54]. Such tropism has been extensively studied in chickens infected with early H5N1 HPAI Gs/GD viruses, which typically show widespread virus replication in vascular endothelium alongside edematous, hemorrhagic, and necrotic cutaneous lesions [44, 45, 48]. Noteworthy, these early H5N1 HPAI Gs/GD viruses are well adapted to and remarkably virulent for chickens [38, 55]. Peracute HPAI virus infections tend to be more endotheliotropic than subacute infections, with subacute infections having more extensive virus replication in parenchymal cells of visceral organs [38, 53]. Therefore, it is unclear why the clade 2.3.4.4A HPAI viruses tested in the present study replicated systemically and extensively in endothelial cells of Pearl guinea fowl but had infrequent endothelial replication and in a limited number of tissues in the other gallinaceous species, even in peracute infections. Interestingly, Pearl guinea fowl not only showed some of the lowest BID_50_ among the gallinaceous species tested, but also some of the shortest MDTs [40], indicating that the virus was well adapted to this host. Further studies are needed to elucidate the mechanisms that determine either restrictive or permissive replication of clade 2.3.4.4A viruses in endothelial cells of different gallinaceous species.

Several studies have shown that different avian species display differential innate immune responses to AI infection [51, 56–60], and that these responses tend to correlate with different pathobiology outcomes [57, 60, 61]. Here, the innate immune responses of chickens and Japanese quail infected with two H5Nx HPAI viruses were compared in order to elucidate any potential link between cytokine responses and clinical or pathological progression of infection. Type II IFN-γ and Th2-type cytokine IL-10 in Japanese quail, and pro-inflammatory IL-6 in chickens, were up-regulated in later clinical stages compared to asymptomatic birds, probably in response to widespread virus replication in parenchymal cells. Previously, Uno et al. found up-regulation of IFN-γ, IL-10, and IL-6 genes in peripheral blood mononuclear cells of Japanese quail collected 24 h after Gs/GD H5N1 HPAI virus challenge and before showing severe neurologic signs around 3 dpc [51]. This supports the idea that the pro-inflammatory cytokine IL-6 is produced early after infection as part of the induced innate immune response and has been associated with the recruitment of inflammatory cells and severe pathology [58, 62, 63]. Similarly, IL-6 was up-regulated in lung and spleen collected from H5 or H7 HPAI virus inoculated chickens [57, 58]. Besides IL-6, innate responses of infected chickens were similar or down-regulated compared to shams, which differs from other studies that find up-regulation in the expression of IFN-α, IFN-γ, and IL-12 in lung and spleen of chickens infected with H5N1 HPAI viruses, H7 HPAI and LPAI viruses, or H9N2 LPAI [56–58, 60, 64, 65]. However, in line with our findings, TLR-7 remained stable in lung of chickens infected with H7 HPAI virus [57], and IL-10 remained stable in lung and spleen of chickens infected with Gs/GD H5 HPAI virus clade 1 [66]. In the present study, chickens appeared to elicit weaker innate immune responses than Japanese quail, suggesting that the lower infectivity and replication of these viruses in chickens may trigger weaker antiviral immune responses. It is worth mentioning that, initially, the innate immune gene expression analysis was performed separately for moribund and dead birds. All genes analyzed had similar mRNA expression levels in moribund and dead birds within each species, confirming that tissue necrosis did not alter cytokine expression. Also, it is worth emphasizing that quantification of innate immune genes was performed on FFPE tissues. Despite the improved isolation methods, RNA obtained from FFPE tissues can be of lower quality and smaller size (less than 200 bp) than fresh tissues due to formalin-induced cross-linking and deterioration of RNA during fixation and storage [67, 68]. Consequently, smaller amplicon sizes are needed for optimal sensitivity [67], with targets in the 70–150-bp being the ideal range [69]. Yet, FFPE tissues have been commonly used for the analysis of RNA expression because of the high availability of FFPE-preserved clinical samples and the direct correlation of these analyses with clinical data [67, 70]. Therefore, although many of our results corresponded with previous data, they should be interpreted with caution.

In conclusion, although the first U.S. clade 2.3.4.4A HPAI viruses in the 2014–2015 outbreak were differently adapted to the six gallinaceous species studied here [39, 40], we observed similar type and severity of histopathological lesions and antigen distribution in those birds that became infected, regardless of virus and species. Asymptomatic or listless infected chickens and Japanese quail lacked microscopic findings, emphasizing the risk of unrecognized virus spread if only passive surveillance is practiced. These viruses appear to have high mortality in minor gallinaceous poultry without prior adaptation, supporting the relevance of minor poultry species in the epidemiology of HPAI as intermediate hosts between wild waterfowl and major commercial gallinaceous poultry. The striking endotheliotropism in Pearl guinea fowl but not the other species calls for further investigation.

## Supplementary information

**Additional file 1. Primers used for the qRRT-PCR of innate immune response genes.**

## Data Availability

The datasets generated and/or analyzed during the current study are included in this published article and its supplementary information files, or are available from the corresponding author on reasonable request.

## Abbreviations

AI: avian influenza
BID_50_: mean bird infectious dose
dpc: day post-challenge
EID_50_: mean egg infectious dose
FFPE: formalin-fixed paraffin-embedded
Gs/GD: A/goose/Guangdong/1/1996
HE: hematoxylin and eosin
HPAI: highly pathogenic avian influenza
IFN: interferon
IL: interleukin
IHC: immunohistochemistry
LPM: live poultry market
LPAI: low pathogenic avian influenza
MDT: mean death time
nt: no tissue
qRRT-PCR: quantitative real-time RT-PCR
SEPRL: Southeast Poultry Research Laboratory
TLR: toll-like receptor
