# Supplementary material for: Pathobiology and innate immune responses of gallinaceous poultry to clade 2.3.4.4A H5Nx highly pathogenic avian influenza virus infection
Source: Vet Res. 2019 Nov 1;50:89. doi: 10.1186/s13567-019-0704-5 (PMC6824115; doi:10.1186/s13567-019-0704-5)
Supplement: Supplementary file 1 — Additional file 1. Primers used for the qRRT-PCR of innate immune response genes. [file 13567_2019_704_MOESM1_ESM.docx]

| Target gene | |  | Primer sequence (5' to 3')^1^ | Length^2^ | Species^3^ | Primer concentration |
| --- | --- | --- | --- | --- | --- | --- |
| Type 1 interferon | IFN-α | For | CCAGCACCTCGAGCAAT | 133 | Ck | 5 µM |
|  |  | Rev | GGCGCTGTAATCGTTGTCT |  | Ck | 5 µM |
|  |  | For | CCTTGCTCCTTCAACGACA | 100 | JQ | 1 µM |
|  |  | Rev | CGCTGAGGATACTGAAGAGGT |  | JQ | 1 µM |
| Type 2 interferon | IFN-γ | For | CTCCCGATGAACGACTTGAG | 111 | Ck | 2.5 µM |
|  |  | Rev | CTGAGACTGGCTCCTTTTCC |  | Ck | 2.5 µM |
|  |  | For | CAACCTTAATGATGGCACGA | 86 | JQ | 5 µM |
|  |  | Rev | CTTTGCGGTGGATTCTCA |  | JQ | 5 µM |
| Th1-type cytokine | IL-12a | For | AAGACCTGAAAACCTACAAGGC | 83 | Ck, JQ | 2.5 µM |
|  |  | Rev | GGCTTGCATCATGTCATCAA |  | Ck, JQ | 2.5 µM |
|  | IL-18 | For | GAAACGTCAATAGCCAGTTGC | 213 | Ck | 5 µM |
|  |  | Rev | TCCCATGCTCTTTCTCACAACA |  | Ck | 5 µM |
|  |  | For | GCAGCGGAATGTACTTCAAC | 94 | JQ | 1 µM |
|  |  | Rev | CTCTTATCTTCTACCTGGACGCTG |  | JQ | 1 µM |
| Th2-type cytokine | IL-10 | For | CATGCTGCTGGGCCTGAA | 94 | Ck | 5 µM |
|  |  | Rev | CGTCTCCTTGATCTGCTTGATG |  | Ck | 5 µM |
|  |  | For | CACAACTTCTTCACCTGCGAG | 96 | JQ | 5 µM |
|  |  | Rev | CATGGCTTTGTAGATCCCGTTC |  | JQ | 5 µM |
| Pro-inflammatory cytokine | IL-6 | For | CTGTTCGCCTTTCAGACCTACC | 219 | Ck | 5 µM |
|  |  | Rev | CATGGTGATTTTCTCTATCCAGTCC |  | Ck | 5 µM |
|  |  | For | CAACCTCAACCTGCCCAA | 202 | JQ | 2.5 µM |
|  |  | Rev | GGAGAGCTTCCTCAGGCATT |  | JQ | 2.5 µM |
| Receptor | TLR-7 | For | TTCTGGCCACAGATGTGACC | 219 | Ck | 5 µM |
|  |  | Rev | CCTTCAACTTGGCAGTGCAG |  | Ck | 5 µM |
|  |  | For | AGATGTTTTCTGGGCAGACG | 177 | JQ | 2.5 µM |
|  |  | Rev | AATGACTTCAACCGGTTACTGG |  | JQ | 2.5 µM |
| Endogenous control | β-actin | For | CTGGCACCTAGCACAATGAA | 123 | Ck, JQ | 2.5 µM |
|  |  | Rev | CTGCTTGCTGATCCACATCT |  | Ck, JQ | 2.5 µM |

^1^ Primers previously described [51].

^2^ Expressed as number of base pairs.

^3^ Ck, chicken. JQ, Japanese quail.
